# Visual acuity outcomes up to 12 years and risk factors for visual impairment in a national cohort of extremely preterm born children – The Extremely Preterm Infants in Sweden Study (EXPRESS)

**DOI:** 10.1111/aos.17525

**Published:** 2025-05-20

**Authors:** Despoina Tsamadou, Karin Källén, Abbas Al‐Hawasi, Ann Hellström, Gerd Holmström, Fatima Pedrosa‐Domellöf, Kristina Tornqvist, Ulrika Kjellström, Lisa B. Thorell, Lena Hellström‐Westas, Andreas Ohlin, Ulrika Ådén, Kerstin Hellgren

**Affiliations:** ^1^ Department of Clinical Neuroscience Karolinska Institutet Stockholm Sweden; ^2^ Astrid Lindgren Children's Hospital Karolinska University Hospital Stockholm Sweden; ^3^ Department of Ophthalmology Örebro University Hospital Örebro Sweden; ^4^ Institution of Clinical Sciences University of Lund Lund Sweden; ^5^ Division of Ophthalmology, Department of Biomedical and Clinical Sciences, Faculty of Medicine Linköping University Linköping Sweden; ^6^ Department of Clinical Neuroscience, Institute of Neuroscience and Physiology, Sahlgrenska Academy University of Gothenburg Gothenburg Sweden; ^7^ Department of Surgical Sciences/Ophthalmology Uppsala University Uppsala Sweden; ^8^ Ophthalmology, Department of Clinical Sciences Umeå University Umeå Sweden; ^9^ Department of Clinical Sciences, Ophthalmology, Skane University Hospital Lund University Lund Sweden; ^10^ Department of Women's and Children's Health Uppsala University Uppsala Sweden; ^11^ Department of Paediatrics, Faculty of Medicine and Health Örebro University Örebro Sweden; ^12^ Department of Women's and Children's Health Karolinska Institutet Stockholm Sweden

**Keywords:** cerebral palsy, cognitive disability, extremely preterm, longitudinal visual outcome, national cohort, perinatal risk factors, retinopathy of prematurity, visual impairment

## Abstract

**Purpose:**

The Extremely Preterm Infants in Sweden Study (EXPRESS) followed a national cohort of extremely preterm born (EPT, i.e. <27 weeks) children until 12 years of age. This study aimed to investigate the longitudinal development of visual acuity (VA) in children born EPT, explore the predictive value of early visual assessments, and evaluate risk factors for visual impairment at the age of 12 years.

**Methods:**

All 462 children born EPT in Sweden during April 2004–March 2007, and surviving to age 6.5 years, and full‐term born matched controls were invited to participate in the 12‐year follow‐up. VA was assessed at 12 years and the results were compared with values at 2.5 and 6.5 years.

**Results:**

At age 12, 332 (72%) EPT survivors and 189 controls were examined. The mean VA in the EPT group was lower than in the control group (1.15, 95%CI [1.12–1.19] vs. 1.33, 95% CI [1.29–1.37]). Fifteen (4.5%) EPT had visual impairment. The examination at age 2.5 failed to identify most of them, while the examination at 6.5 could predict the final visual outcome at 12. Risk factors for visual impairment were low gestational age, moderate and severe intraventricular haemorrhage, treatment‐requiring retinopathy of prematurity, cerebral palsy, and cognitive disability.

**Conclusion:**

In this national cohort, the VA outcome at age 12 was lower in children born EPT than full‐term controls. As eye examination at 2.5 years did not reliably identify visual impairment, clinical risk factors should be considered in the screening of children born EPT to early identify the visually impaired.

## 1 INTRODUCTION

Thanks to advances in neonatal care, an increasing number of children born extremely preterm (EPT) survive, though encountering challenges related to health and quality of life (Ancel et al., 2015; Norman et al., 2019). The multifaceted consequences of EPT birth in infancy and later life are thoroughly described (Johnson & Marlow, 2017; McBryde et al., 2020; Pierrat et al., 2021). The risk of visual impairment (VI) is high in children born preterm, the underlying causes being both ocular and cerebral (Blencowe et al., 2013; Jacobson & Dutton, 2000; O'Connor & Fielder, 2008). Several longitudinal studies have shown that low gestational age (GA) and birth weight, retinopathy of prematurity (ROP), and ROP treatment affect visual function and ocular development (Fielder et al., 2015; Holmström & Larsson, 2008; Venkataraman et al., 2023). Furthermore, brain white matter damage, commonly seen in infants born preterm, has been shown to cause cerebral visual impairment (CVI) (Al‐Abaiji et al., 2024; Dutton, 2013; Fazzi et al., 2009). CVI is an umbrella diagnosis covering visual perceptual deficits and usually subnormal visual acuity (VA), which cannot be fully explained by ocular abnormalities (Sakki et al., 2018).

However, most available studies are based on preterm‐born populations surviving before recent advances in neonatal care (Fieß et al., 2023; Johnson & Marlow, 2017). In addition, studies based on children born EPT and receiving modern neonatal care and treatment modalities for ROP have only had their ophthalmological outcomes reported until early school age (Chapron et al., 2022; Larsson et al., 2023). Many previous studies have also used relatively small, selected samples. Thus, there is a lack of population‐based studies of EPT survivors after modern neonatal care that have followed visual development at different ages and further on into pre‐adolescence.

Due to the risk of VI in children born EPT, several follow‐up screening programmes for visual development have been established, mainly based on the presence of ROP (AAPOS, n.d.; NICE Recommendations, n.d.; Recommendations Francaises Pour le Depistage de la Retinopathie des Prematures, n.d.; SWEDROP, n.d.). Determining the optimal ages for reliable eye examinations is though challenging, as this group of children is also burdened with neurological and cognitive disabilities, as well as behavioural disorders, making the execution and results of eye examination at a young age uncertain (Franz et al., 2018; Pascal et al., 2018).

The Extremely Preterm Infants in Sweden Study (EXPRESS) is a national multidisciplinary prospective study with the aim to investigate survival and morbidities in all infants born in Sweden before GA 27 weeks, from April 2004 to March 2007 (EXPRESS Group, 2010). Ophthalmological assessments were performed at 2.5 (corrected age), 6.5, and 12 years of age (Hellgren et al., 2016; Holmström et al., 2014). As previously published, at 6.5 years of age, 38% had major eye and visual problems, of whom 4.8% were visually impaired according to World Health Organization criteria. The prevalence was strongly related to the degree of prematurity, treatment‐requiring ROP, bronchopulmonary dysplasia, and cerebral palsy (CP) (Hellström et al., 2018). The research group also found a high rate of various features of CVI in a subgroup of the cohort using a questionnaire (Hellgren et al., 2020). Although the frequency of visually impaired was low in this subgroup, a negative association between VA and CVI was found.

Children born EPT often have complex visual impairments that may be difficult to detect at an early age, but which nevertheless require early adaptations and interventions. VA is the common denominator in assessments of visual function and is often used as a primary criterion for accessing a low vision clinic. Therefore, we chose to focus on VA when studying the visual outcome in our cohort.

The present study aimed to (1) investigate the longitudinal development of VA in an extremely preterm population (EXPRESS) from 2.5 to 12 years of age, (2) explore the predictive value of early visual assessments for VA at 12 years of age, and (3) investigate the associations between perinatal factors, cognitive disability (CD), cerebral palsy (CP) and the VA at 12 years.

## 2 MATERIALS AND METHODS

### 2.1 Study population

The EXPRESS cohort included 707 liveborn EPT infants aged below 27 gestational weeks (EXPRESS Group et al., 2009). At 6.5 years, 462 survivors were eligible for visual examination (Hellgren et al., 2016). These children were also invited to participate in the present 12‐year follow‐up study. A group of control children, born full‐term and matched for age, sex, postal code, and maternal country of origin, was included in the 6.5‐year follow‐up and was invited to participate in the follow‐up at age 12 years (Hellgren et al., 2016).

### 2.2 Methods

The ophthalmological examination was performed at 12 years ±6 months for the participating children born EPT and the controls, according to the same study protocol at all seven university hospitals in Sweden. The medical records of EPT children not attending the 12‐year follow‐up were reviewed. VA was measured monocularly and binocularly at 3 m, with habitual correction, using linear Sloan letter optotype in logarithmic progression, and was registered on a decimal scale. The best measurable VA was 2.0; a minimum of four out of five letters had to be identified in each line. Near vision was examined with the standardized single‐letter near‐vision optotype KM (Moutakis et al., 2004).

Binocular VA, or the VA of the better eye, was used as the best estimate. When distance VA values were lacking, near‐vision measurements were used when available. To follow the visual development in the EPT group, we compared the VA values and VI categories at 12 years with those assessed at previous follow‐ups at 2.5 and 6.5 years. Only examinations obtained within 1 year before and after the target age were included.

At the 2.5‐year follow‐up, the visual assessment was based on a single Lea Hyvärinen optotype, toy, and light. No VI (class 0) was defined as being able to see the optotype or toy, VI (class 1) as only fixating light, and blindness (class 2) as not seeing the light (Holmström et al., 2014). At the 6.5‐year follow‐up, VA was examined using a linear symbol optotype (Lea Hyvärinen), and VI was defined according to WHO criteria (Hellgren et al., 2016). Thus, a 5‐class scale according to the WHO classification for VI was applied to both 6.5‐ and 12‐year follow‐ups: class 0: no VI (VA ≥ 0.5); class 1: mild VI (VA ≥ 0.33 and <0.5); class 2: moderate VI (VA ≥ 0.1 and <0.33); class 3: severe VI (VA ≥ 0.05 and <0.1); class 4: blindness (VA <0.05) (World Health Organization, 2019). Children categorized into classes 1–4 were defined as visually impaired.

We analysed perinatal data, including GA, intraventricular haemorrhage (IVH), ROP stage, and ROP treatment, and categorized data from follow‐ups at 2.5 and 6.5 years of age, comprising CP, CD, and VI. We included data collected during ophthalmological examinations for the controls at ages 6.5 and 12 years. The methods used to collect those data and classification of IVH, ROP, CP, and CD are described in previous publications from this cohort (Hellgren et al., 2016; Holmström et al., 2014; Serenius et al., 2013; Serenius et al., 2016).

### 2.3 Statistical analysis

Chi‐square tests were used to compare background data between EPT children included and not included in the 12‐year follow‐up. VA values were compared between EPT children and controls using the Mann–Whitney U test, having first excluded blind children. We used linear mixed effects models to compare the VA development from 6.5 to 12 years between the EPT and control groups. The Fisher's exact test investigated associations between early risk factors (GA, IVH, ROP, ROP treatment, CP, CD, and VI) and VI at 12 years. *p*‐values < 0.05 were considered statistically significant. Statistical analyses were performed using IBM SPSS Statistics, version 27.0 (Armonk, NY: IBM Corp, USA) and Gauss (Aptech Systems Inc., Arizona, USA).

### 2.4 Ethics

The Ethics Review Board in Lund, Sweden, approved the study according to the Tenets of the Declaration of Helsinki. Informed consent was obtained from the caregivers of all participants, and ethical approval was given to review the medical records of EPT children who did not attend.

## 3 RESULTS

### 3.1 Participants

At the 12‐year follow‐up, 308 of 462 (67%) EPT children attended. The retrieval of medical records of EPTs who did not attend this visit supplied ophthalmological data for 24 additional children in this age group. Thus, 332 EPT children were included, the participation rate being 72%. In previous follow‐ups, the participation rates were 84% at 2.5 years and 88% at 6.5 years.

At the 6.5‐year follow‐up, 300 controls attended and 189 of those attended at the 12‐year follow‐up. No controls were examined at the 2.5‐year follow‐up.

No data were obtained from 130 EPT children; they were considered dropouts. There were no statistically significant differences between the included EPT children and dropouts regarding GA, IVH, and ROP. However, moderate and severe CP and CD, and VI at ages 2.5 and 6.5 years were significantly overrepresented among dropouts (Table 1).

**TABLE 1:** Neonatal characteristics and scores at 2.5‐and 6.5‐ year follow‐up, respectively, in relation to the availability of 12‐year eye data in the EPT group.

	12‐year data available		12‐year data not available		*p*‐value*
	*N* = 332 (72%)		*N* = 130 (28%)		
	*n*	%	*n*	%	
GA					0.138
22w	4	1.2	1	0.8	
23w	36	10.8	11	8.5	
24w	62	18.7	26	20.0	
25w	120	36.1	34	26.2	
26w	110	33.1	58	44.6	
ROP stage					0.593
0	90	27.1	39	30.0	
1–2	123	37.0	53	40.8	
3–4	119	35.8	38	29.2	
ROP treatment	63	19.0	23	17.7	
IVH grade					0.417
0	203	61.1	85	65.4	
1–2	96	28.9	29	22.3	
3–4	33	9.9	13	10.0	
VI at 2.5 years					0.038
Yes	6	1.8	5	3.8	
No	281	84.6	97	74.6	
Not known	45	13.6	28	21.5	
CD at 2.5 years					0.023
No or mild	270	81.3	94	72.3	
Moderate or severe	58	17.5	30	23.1	
Not known	4	1.2	6	4.6	
CP at 2.5 years					0.027
No or mild	309	93.1	112	86.2	
Moderate or severe	19	5.7	12	9.2	
Not known	4	1.2	6	4.6	
VI at 6.5 years					<0.001
Yes	14	4.2	8	6.2	
No	311	93.7	108	83.1	
Not known	7	2.1	14	10.8	
CD at 6.5 years					<0.001
No or mild	233	70.2	76	58.5	
Moderate or severe	94	28.3	38	29.2	
Not known	5	1.5	16	12.3	
CP at 6.5 years					<0.001
No or mild	317	95.5	107	82.3	
Moderate or severe	10	3.0	7	5.4	
Not known	5	1.5	16	12.3	

Abbreviations: CD, cognitive disability; CP, cerebral palsy; EPT, extremely preterm; GA, gestational age; IVH, intraventricular haemorrhage; ROP, retinopathy of prematurity; VI, visual impairment.

* *p*‐value for homogeneity obtained by Chi‐square analyses.

### 3.2 Visual acuity at 12 years

**FIGURE 1:**
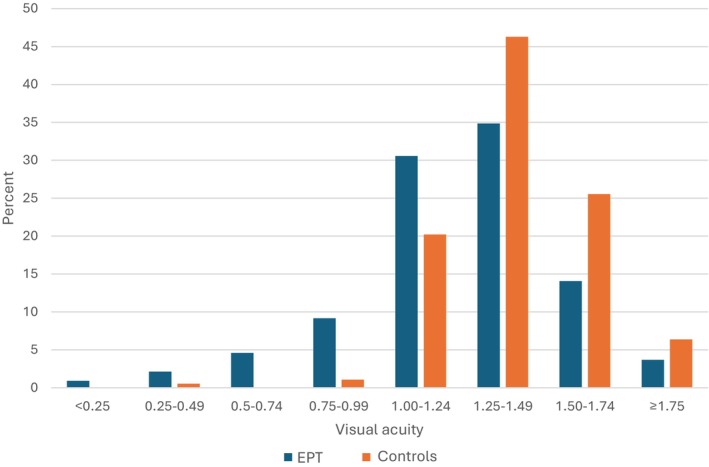
Distribution of VA values at age 12 years for EPT and controls. Median VA in EPT group 1.25 (IQR 1.00–1.25). Median VA in control group 1.25 (IQR 1.25–1.60). *p*‐value for different distributions <0.001 (Mann–Whitney *U* test). VA indicates visual acuity. EPT indicates extremely preterm.

Based on best estimate values and also after excluding blind children, a difference was found in mean VA between EPT children (1.15, 95% CI [1.12–1.19]) and controls (1.33, 95% CI [1.29–1.37]) at the age of 12. The median VA (1.25) was the same in the EPT and control groups, but a statistically significant difference in the distribution of VA values was noted, with skewness towards lower VA in the EPT group (Figure 1).

**FIGURE 2:**
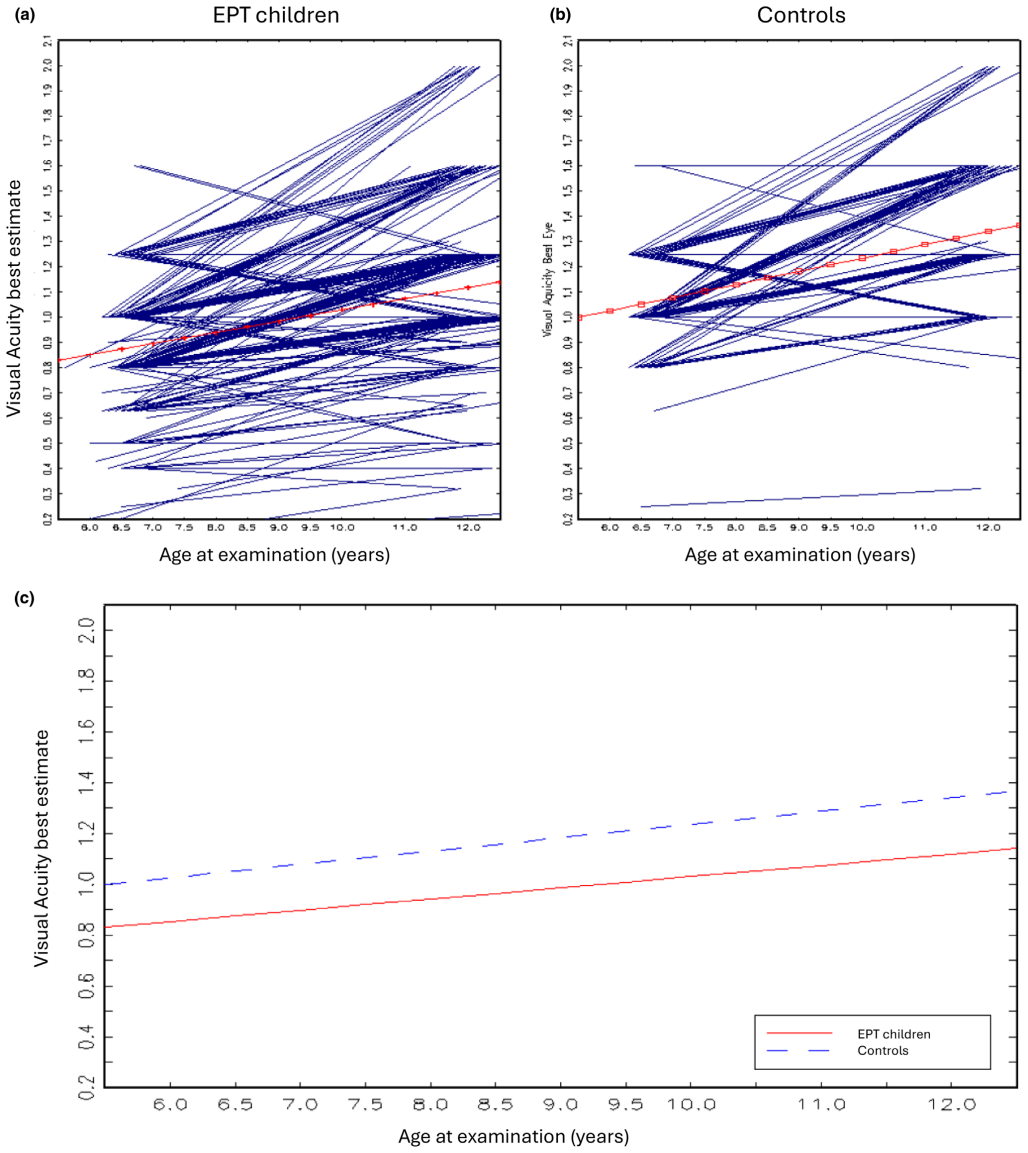
Development of visual acuity from 6.5 to 12 years in EPT and controls. (a and b) Trajectories of development of visual acuity, best estimate values for every participant between ages 6.5 and 12 years for EPT and full‐term controls. (c) Development of mean visual acuity between ages 6.5 and 12 years, comparison between EPT and full‐term controls. EPT indicates extremely preterm. *p*‐value for level difference: 0.014, *p*‐value for linear difference: 0.0975.

We followed the development of VA from 6.5 to 12 years of age for both EPT children and controls. VA values improved in both groups, though the mean VA was persistently significantly lower in the EPT group (Figure 2).

### 3.3 Visual impairment from 2.5 to 12 years

Among the 332 EPT children examined at 12 years of age, 15 (4.5%) had VI, of whom four had mild VI, six had moderate VI, and five were blind. The rates of VI (classes 1–4) were 3.1% at 2.5 years and 8.8% at 6.5 years (Hellgren et al., 2016; Holmström et al., 2014). The frequencies of VI at the different ages are presented in Table 2 (a, b).

**TABLE 2:** VI‐scores assessed at different ages. (a) VI‐scores at age 6.5 and 12 years, respectively, in relation to VI‐scores at 2.5 years. (b) VI‐scores at age 12 years in relation to VI‐scores at 6.5 years.

(a)													
	Total	VI‐class^a^ at 6.5 years						VI‐class^a^ at 12 years					
		0	1	2	3	4	Not known^b^	0	1	2	3	4	Not known^b^
Total (*n*)	462	375	13	10	2	8	54	317	4	6	0	5	130
VI‐class^a^ at 2.5 years													
0	372	*321*	10	6	1	0	34	*273*	3	3	0	0	93
1	6	2	*0*	*1*	0	2	1	2	*0*	*1*	0	0	3
2	3	0	0	0	*0*	*3*	0	0	0	0	*0*	*2*	1
Not known^b^	81	52	3	3	1	3	*19*	42	1	2	0	3	*33*

(b)							
	Total	VI‐class^a^ at 12 years					
		0	1	2	3	4	Not known^b^
Total (*n*)	462	317	4	6	0	5	130
VI‐class^a^ at 6 years							
0	375	*295*	0	0	0	0	80
1	13	6	*2*	1	0	0	4
2	10	2	2	*4*	0	0	2
3	2	0	0	0	*0*	0	2
4	8	0	0	1	0	*5*	2
Not known^b^	54	14	0	0	0	0	*40*

^a^ VI‐class according to WHO definition.

^b^ Not known: no data on vision.

The number of visually impaired children born EPT at the 6.5‐year and 12‐year follow‐ups in relation to the 2.5‐year follow‐up is presented in Table 2a. As shown there, 372 (80%) children had no VI at 2.5 years; of those, 10 had mild, six had moderate, and one had severe VI at 6.5 years, while at 12 years, three had mild and three had moderate VI. Thus, six children with VI at 12 years were assessed as having normal vision at 2.5. The three children assessed as blind at 2.5 years were still evaluated as blind at 6.5 years; at 12 years, one did not attend, and the other two were still blind.

At age 2.5 years, no visual data could be obtained for 81 EPTs, of whom 63 did not attend the eye examination, and 18 were challenging to examine. Of the 81 children who were not assessed, 10 (12%) were visually impaired at the 6.5‐year follow‐up, and at the 12‐year follow‐up, three were blind, two had moderate VI, and one had mild VI. Among the 18 EPTs challenging to examine at 2.5 years, two had mild VI, and two had moderate VI at 6.5 and 12 years. Thus, four children with VI were missed at 2.5 years due to non‐cooperation. When analysing whether they had other risk factors leading to suspected visual deficits, we found proportionally higher rates of moderate/severe CD and ROP treatment in this dropout group. In contrast, strabismus, IVH, and CP rates were similar in the attending EPT group.

From age 6.5 to 12 years, most EPT children remained in the same VI category. Eleven children (33%), evaluated as visually impaired at 6.5 years and assessed at 12 years, had improved their vision at the 12‐year follow‐up. The dropout rate was higher at 12 years, so 10 out of 33 children (30%) evaluated as visually impaired at 6.5 years were not assessed at the 12‐year follow‐up (Table 2b).

Only one control child had moderate VI at 6.5‐ and at 12‐year follow‐ups.

### 3.4 Visual acuity at 12 years related to risk factors

We found a significant negative relation between GA and VI (*p* = 0.033). The proportion of children with VI per GA in weeks was one of four (25%) for GA 22, three of 36 (8.3%) for GA 23, four of 62 (6.5%) for GA 24, six of 120 (5%) for GA 25, and one of 110 (0.9%) for GA 26. Of the 33 EPT children with IVH stage 3–4, seven (21%) had VI. Of ROP‐treated children, 17.5% were visually impaired, while no VI was found among those with severe ROP without treatment. Most EPT born children with VI had received ROP treatment (11 of 15; 73%).

A CP and/or CD diagnosis was strongly related to VI at 12 years. A majority of children (60%) diagnosed with moderate/severe CP at age 6.5 years and almost half (44%) of those with moderate/severe CP at age 2.5 years had VI at 12 years. Two‐thirds of the visually impaired EPT were assessed as having a moderate or severe CD at both 2.5‐ and 6.5‐year follow‐ups, and these correlations were statistically significant (*p* < 0.001).

The distribution of visually impaired EPT children in relation to studied risk factors is illustrated in Table 3.

**TABLE 3:** Neonatal characteristics and scores at 2.5‐ and 6.5‐year follow‐up in relation to visual impairment at 12 years in the EPT group.

	Total	Visual impairment at 12 years				*p*‐value*
		Yes		No		
	*N*	*n*	%	*n*	%	
Total, all children	332	15	4.5	317	95.5	
GA						0.033
22w	4	1	25.0	3	75.0	
23w	36	3	8.3	33	91.7	
24w	62	4	6.5	58	93.5	
25w	120	6	5.0	114	95.0	
26w	110	1	0.9	109	99.1	
ROP stage						0.022
0	90	2	2.2	88	97.8	
1–2	123	2	1.6	121	98.4	
3–4	119	11	9.2	108	90.8	
Treatment	63	11	17.5	52	82.5	<0.001**
IVH grade						<0.001
0	203	6	3.0	197	97.0	
1–2	96	2	2.1	94	97.9	
3–4	33	7	21.2	26	78.8	
VI at 2.5 years						<0.001
Yes	6	3	50.0	3	50.0	
No	281	6	2.1	275	97.9	
Not known	45	6	13.3	39	86.7	
CD at 2.5 years						<0.001
No or mild	270	4	1.5	266	98.5	
Moderate or Severe	58	11	19.0	47	81.0	
Not known	4	0	0.0	4	100	
CP at 2.5 years						0.001
No or mild	319	11	3.4	308	96.6	
Moderate or severe	9	4	44.4	5	55.6	
Not known	4	0	0.0	4	100	
VI at 6.5 years						<0.001
Yes	23	15	65.2	8	34.8	
No	295	0	0.0	295	100	
Not known	14	0	0.0	14	100	
CD at 6.5 years						<0.001
No or mild	233	3	1.3	230	98.7	
Moderate or severe	94	12	12.8	82	87.2	
Not known	5	0	0.0	5	100	
CP at 6.5 years						<0.001
No or mild	317	9	2.8	308	97.2	
Moderate or severe	10	6	60.0	4	40.0	
Not known	5	0	0.0	5	100	

Abbreviations: CD, cognitive disability; CP, cerebral palsy; EPT, extremely preterm; GA, gestational age; IVH, intraventricular haemorrhage; ROP, retinopathy of prematurity; VI, visual impairment.

* Obtained from Fisher–Freeman–Halton Exact test.

** Versus no treatment.

Further analysis of risk factors for each visually impaired EPT child at 12 years is supplied in the supplemental Table S1. In most cases, treatment‐requiring ROP and severe brain damage co‐existed.

## 4 DISCUSSION

This prospective longitudinal study followed the development of VA from early childhood to pre‐adolescence in a national cohort of children born EPT. The differences in mean VA and the distribution of VA values between EPT and controls at age 12 years demonstrate that in accordance with previous Swedish long‐term studies, pre‐adolescents born EPT do not manage to catch up with their full‐term peers (Holmström & Larsson, 2008; Larsson et al., 2005; Pétursdóttir et al., 2020).

At the age of 2.5 years, a high proportion of children could not participate in the eye examination (17%). Among them, there was an overrepresentation of children with moderate and severe VI, discovered at the 6.5‐year follow‐up of our study. There is a great need to identify children born EPT with VI early in development to be able to provide interventions. However, the existing follow‐up screening programmes for preterm‐born children show high discrepancies in inclusion criteria and examination intervals. Some focus predominantly on the diagnosis or treatment of ROP (AAPOS), others on GA (France) or/and perinatal risk factors (UK, Sweden) (AAPOS, n.d.; NICE recommendations, n.d.; Recommendations Francaises Pour le Depistage de la Retinopathie des Prematures n.d.; SWEDROP, n.d.).

Still, VI observed at a young age was a definite risk factor for VI at 12 years: 50% of the visually impaired at 2.5 and 65% at 6.5 had VI at 12 years. On the contrary, we discovered six children with mild or moderate VI among those assessed as having normal vision at age 2.5 years. Two national prospective studies previously reported similar observations (Leversen et al., 2012; Marlow et al., 2005). It is therefore reasonable to be cautious in interpreting early visual examination. The findings of the 6.5‐year follow‐up are, on the other hand, more consistent with those of the 12‐year follow‐up. By what Hellgren et al. hypothesized, VA improved with age in some children who tested as having a level of vision permitting a driver's licence (Hellgren et al., 2016) Nevertheless, there is still a significant proportion of visually impaired children in this population who, considering the co‐morbidities in EPT, grow into adulthood with multiple life‐long disabilities.

Investigating risk factors for VI, we found a strong relation between VI and the degree of prematurity, confirming findings of the previous EXPRESS study at 6.5 years, and comparable to other studies (Blencowe et al., 2013; Hellgren et al., 2016). Moreover, CP, CD, and treatment for ROP were dominant risk factors affecting the final visual outcome, in line with a recent Swedish publication focusing on the tiniest EPTs (Hellström et al., 2022).

The severity of ROP, and most significantly, the treatment for ROP were strongly related to VI. This agrees with previous results in the 6.5‐year follow‐up of EXPRESS, where major visual problems were linked to treatment‐requiring ROP and numerous other studies (Blencowe et al., 2013; Hellström et al., 2018). Nevertheless, a recent national French study showed that ROP was associated with other ophthalmological morbidities but not VI (Chapron et al., 2022). This might be explained by a wider inclusion range in that study, where the less premature babies dominated. Moreover, a publication of the ELGAN study showed an association between ROP and brain damage (Allred et al., 2014). As new, less destructive treatment modalities for ROP may have favourable anatomical outcomes, further studies are necessary to show to what extent ROP and its treatment, or the changes in visual pathways, account for VI (Robitaille, 2024; Sternberg & Durrani, 2018).

Also, IVH was associated with an increased risk for VI, and the proportion of VI among children with severe IVH was 21%, compared with only 2% among those with mild IVH. This is consistent with a recent meta‐analysis, where the authors specify that children with IVH grades 1–2 had an increased risk for VI, and those with grades 3–4 had a markedly higher risk (Rees et al., 2022).

CP and CD were strongly associated with VI at 12 years of age. CP is a well‐studied risk factor for multiple visual impairments, as described by a recent review (Crotti et al., 2024). In our study, 60% of EPT children with moderate–severe CP at 6.5 years and 44% at 2.5 years had VI in pre‐adolescence. It has been speculated that the grade of CP cannot be optimally defined at a younger age, and this might explain the discrepancy (Serenius et al., 2016). Indeed, we observed a higher affinity between CP at 6.5 years and VI. CD, both at ages 2.5 and 6.5 years, was equally and positively related to VI. This is comparable to an observation from a smaller EPT cohort, showing that CD at 2.5 years predicted school‐age visual problems (Hreinsdottir et al., 2018). As previously presented by Allred et al., common inflammatory and angiogenetic mechanisms behind brain damage and ROP in EPT can explain the near association between CP, CD, and VI. Indeed, when we scrutinized risk factors for each of the 15 visually impaired children (Table S1), we found that in most cases, there were several plausible causes for their VI, both ocular and cerebral.

The incidence of VI at 12 years (4.5%) was lower than that at 6.5 years (8.8%), but the higher rate of dropouts should be considered. We suspect that the incidence of VI at 12 years may be even higher as severely visually impaired pre‐adolescents can be found among dropouts. Indeed, the rates of previously confirmed CP, CD, and VI were higher in the dropout group compared with the studied group. Since we have shown here that these risk factors were strongly related to VI, it would be logical to expect that children with multiple disabilities might have declined further eye examinations, thus not attending our follow‐up; this is a previously reported pattern (Molloy et al., 2013).

The main strength of this study is its duration. The ability to follow longitudinally, from birth to adolescence, and register numerous variables in a national cohort, is unique to Sweden. Few studies with a similar design are found in the literature, and none has reported visual outcomes on consecutive follow‐ups for such a large age span. Another strength is its national basis and coverage (72%), making the studied cohort representative of the national population. During the design of this study, we considered whether visual examination should be done using habitual or best correction; we decided on the former. Even if measurements with habitual correction underestimate the visual potential of those EPT adolescents, they reflect the quality of their vision in everyday life. A possible limitation of our study was that some measurements were not performed according to the study protocol; however, the results were comparable since the visual examination methods are uniform throughout Sweden.

Although the primary purpose of our study was to describe the longitudinal visual outcome of extremely preterm birth, we came across an unexpected finding: the visual examination at age 2.5 years, a current routine in Swedish clinical practice, failed to identify most children (10 out of 15) with VI at 12 years in this cohort. The rather low predictive value of the 2.5‐year follow‐up indicates a need to redefine suitable age and examination methods for ophthalmic follow‐up after EPT birth. We therefore advocate a re‐evaluation of non‐cooperating children within 6 months. In addition, we suggest that screening guidelines should include, besides low GA and ROP treatment, also severe IVH, CP, and CD in the follow‐up of this vulnerable population.

## 5 CONCLUSION

By the age of 12, children born EPT did not catch up with their full‐term peers in VA. Further, the 12‐year follow‐up reflected the results at age 6.5, but not at age 2.5. The 2.5‐year follow‐up failed to identify VI in two‐thirds of the children born EPT who were visually impaired at 12 years. Low GA, treatment‐requiring ROP and moderate to severe IVH, CD, and CP are important risk factors for later VI. In the ophthalmological follow‐up of children born EPT, accounting for risk factors and individualizing controls in infants with questionable results is essential for the timely detection of VI.

## AUTHOR CONTRIBUTIONS

Drs Hellgren and Källén had full access to all the data in the study and take responsibility for the integrity of the data and the accuracy of the data analysis. Study concept and design: All authors. Acquisition, analysis, or interpretation of data: All authors. Drafting of the manuscript: Tsamadou, Hellgren. Critical revision of the manuscript for important intellectual content: All authors. Statistical analysis: Källén. Obtained funding: Hellgren, Tsamadou, Hellström, Åden, Thorell, Källén. Administrative, technical, or material support: All authors. Study supervision: All authors.

## FUNDING INFORMATION

This research was funded by the Swedish Research Council, grant numbers 2014‐03908, 2020‐01092, 2006‐3858, 2009‐4250, the Stockholm County Council and Karolinska Institutet (ALF‐20160227, FoUI 947 257, 949 327, 951 235, 952 920, 960 250), Government grants under the ALF agreement (ALFGBG‐717971 and ALFGBG‐971188), Knut and Alice Wallenberg Foundation Clinical Scholars, the Sigvard and Marianne Bernadotte Research Foundation for Children Eye Care, Ögonfonden.

## ROLE OF THE FUNDER/SPONSOR

The funders had no role in the design and conduct of the study; collection, management, analysis, and interpretation of the data; preparation, review, or approval of the manuscript; and decision to submit the manuscript for publication.

## PREVIOUS PRESENTATIONS

A part of the results has previously been presented at the EPOS Annual meeting in Leuven, Belgium in October 2023 and at the NPOG meeting in Reykjavik, Iceland in May 2024.

## Supporting information


Table S1.
