# Supplementary material for: Visual acuity outcomes up to 12 years and risk factors for visual impairment in a national cohort of extremely preterm born children – The Extremely Preterm Infants in Sweden Study (EXPRESS)
Source: Acta Ophthalmol. 2025 May 20;104(1):65–74. doi: 10.1111/aos.17525 (PMC12803563; doi:10.1111/aos.17525)
Supplement: Supplementary file 1 — Table S1. [file AOS-104-65-s001.docx]

Table S1. Visually impaired EPT children at 12 years in relation to risk factors

| VI 12 yrs | GA | ROP stage | ROP treat | IVH grade | VI 2.5yrs | CP 2.5yrs | CD 2.5yrs | VI 6.5 yrs | CP 6.5yrs | CD 6.5 yrs |
| --- | --- | --- | --- | --- | --- | --- | --- | --- | --- | --- |
| 1 | 23 | 3 | yes | 4 | 0 | 3 | 3 | 2 | 3 | 4 |
| 1 | 25 | 3 | yes | 3 | 0 | 1 | 2 | 1 | 1 | 4 |
| 1 | 24 | 3 | yes | 2 | dropout | 1 | 3 | 1 | 1 | no value |
| 1 | 22 | 3 | yes | 0 | 0 | 1 | 4 | 2 | 1 | 4 |
| 2 | 25 | 0 | no | 0 | 0 | 1 | 4 | 2 | 3 | 1 |
| 2 | 23 | 3 | yes | 3 | 0 | 3 | 2 | 2 | 3 | 4 |
| 2 | 26 | 0 | no | 2 | no-coop | 1 | 4 | 2 | 2 | no value |
| 2 | 25 | 2 | no | 3 | 0 | 1 | 1 | 1 | 1 | 1 |
| 2 | 24 | 3 | yes | 0 | no-coop | 1 | 3 | 2 | 1 | 4 |
| 2 | 25 | 3 | yes | 0 | 1 | 1 | 4 | 4 | 1 | no value |
| 4 | 23 | 5 | yes | 0 | 2 | 1 | 1 | 4 | 1 | no value |
| 4 | 25 | 4 | yes | 0 | dropout | 1 | 3 | 4 | 1 | no value |
| 4 | 25 | 2 | no | 4 | dropout | 4 | 4 | 4 | 3 | 3 |
| 4 | 24 | 3 | yes | 4 | dropout | 1 | 4 | 4 | 2 | no value |
| 4 | 24 | 3 | yes | 4 | 2 | 4 | 4 | 4 | 3 | no value |

Abbreviations

EPT: extremely preterm

VI: visual impairment class according to WHO definition

GA: gestational age in weeks

ROP: retinopathy of prematurity, stages 1-5

ROP treat: treated for retinopathy of prematurity, yes/no

IVH: intraventricular haemorrhage, grades 1-4

CP: cerebral palsy, 1-2: no or mild, 3-4: moderate-severe

CD: cognitive disability, 1-2: no or mild, 3-4: moderate-severe

no-coop: non-cooperating at follow-up

dropout: not attending follow-up
